# Acute myocardial infarction in a patient with hypofibrinogenemia: a case report

**DOI:** 10.1186/1752-1947-5-582

**Published:** 2011-12-19

**Authors:** Fathia Mghaieth, Habiba Mizouni, Sihem Mbarki, Jihen Ayari, Ramy Trabelsi, Nidhal Ben Moussa, Mohamed Sami Mourali, Emna Mnif, Rachid Mechmeche

**Affiliations:** 1 Service of Cardiology, Rabta University Hospital, Faculty of Medicine, University of Tunis El Manar, Tunis, Tunisia; 2 Service of Radiology, Rabta University hospital. Faculty of Medicine, University of Tunis El Manar, Tunis, Tunisia

## Abstract

**Introduction:**

Congenital fibrinogen deficiency is a rare coagulation disorder usually responsible for hemorrhagic diathesis. However, it can be associated with thrombosis and there have been limited reports of arterial thrombotic complications in these patients.

**Case presentation:**

A 42-year-old Tunisian man with congenital hypofibrinogenemia and no cardiovascular risk factors presented with new onset prolonged angina pectoris. An electrocardiogram showed features of inferior acute myocardial infarction. His troponin levels had reached 17 ng/L. Laboratory findings confirmed hypofibrinogenemia and ruled out thrombophilia. Echocardiography was not useful in providing diagnostic elements but did show preserved left ventricular function. Coronary angiography was not performed and our patient did not receive any anticoagulant treatment due to the major risk of bleeding. Magnetic resonance imaging confirmed myocardial necrosis. Our patient was managed with aspirin, a beta-blocker, an angiotensin-converting enzyme inhibitor and statin medication. The treatment was well tolerated and no ischemic recurrence was detected.

**Conclusion:**

Although coronary thrombosis is a rare event in patients with fibrinogen deficiency, this condition is of major interest in view of the difficulties observed in managing these patients.

## Introduction

Hypo- and afibrinogenemia are rare congenital coagulation disorders with an estimated incidence of one in 500,000 births [1]. They are usually responsible for an increased bleeding tendency. Although uncommon, some cases of venous and arterial thrombotic events associated with fibrinogen deficiency have been reported in the literature [1-9]. The mechanism of this apparently paradoxical thrombotic tendency, which has long remained problematic, has now been partially clarified [10].

## Case presentation

We report the case of a 42-year-old Tunisian man, born out of a consanguineous marriage, who had no cardiovascular risk factors and was followed-up for hypofibrinogenemia diagnosed three years previously due to bleeding after dental care. He had no known family history of fibrinogen deficiency. He was admitted for an acute typical anginal pain which occurred at rest and continued for several hours.

**Figure 1:**
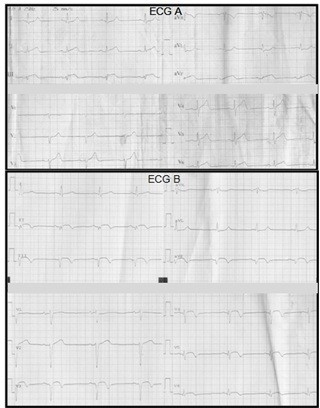
**ECG findings**. **(A) **Electrocardiogram on admission. ST-segment elevation in DIII, aVF and ST- segment depression in DI, aVL. **(B) **Electrocardiogram on the seventh day. Q wave in inferior leads and apicolateral subepicardial ischemia.

**Figure 2:**
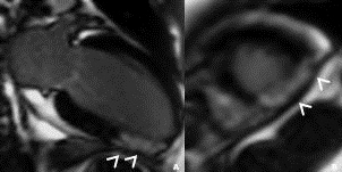
**Contrast-enhanced inversion-recovery magnetic resonance study on (A) long axis and (B) short-axis images during the subacute phase (day five)**. Almost complete transmural enhancement in the infero-apicolateral wall (arrowheads).

On physical examination he had no fever, his blood pressure was 120/70 mmHg, his heart rate was 65 beats per minute and cardiopulmonary auscultation was normal. An electrocardiogram (ECG) on admission, seven hours after the onset of pain (Figure 1A) showed an elevation of the ST segment in inferior leads and an ST-segment depression in DI, aVL. His troponin I and creatine phosphokinase levels were elevated to 17 ng/L and 820IU/L respectively. There was no sign of inflammation; his C-reactive protein level was 5 mg/L and white blood cell count, 7000 cells/mL. Bleeding-related tests were carried out. His fibrinogen level was 0.3 g/L using the Von Clauss method, and < 0.16 g/L using the immunological method. His activated partial thromboplastin time was > 120 seconds (control: 32 seconds), prothombin activity < 10% and thrombin time > 120 seconds (control: 13 seconds). His platelet count (242,000/mm^3^) and bleeding time (140 seconds) were normal. A transthoracic echocardiography estimated his left ventricular ejection fraction at 61% without evidence of segmental motion abnormalities. A coronary angiography was not performed in view of the major risk of bleeding. Cardiac magnetic resonance imaging (MRI) on day 5 (Figure 2) showed the presence of almost complete transmural enhancement of the apicolateral segment. It is noteworthy that an etiological investigation for acute myocardial infarction in a young adult was otherwise negative; our patient did not have any protein C or protein S or an antithrombin deficiency; there was neither a Factor V Leiden nor prothrombin G20210 mutation, no anti-phospholipid antibodies (lupus type v inhibitor, anticardiolipin or anti-2-glycoprotein-1 antibodies) were detected and his plasma homocysteine level was normal. His fasting glucose (0.9 g/L) and hemoglobin A1C (4.7%) levels, lipid profile (cholesterol: 1.8 g/L; high density cholesterol: 0.47 g/L; triglycerides: 1.6 g/L; low density cholesterol (Friedewald formula): 1.0 g/L) and liver function tests were also normal. Transesophageal echocardiography ruled out an emboligenic disease. Our patient was managed with aspirin, atenolol, captopril and atorvastatin but did not receive any anticoagulant treatment. He did not experience any angina recurrence.

**Figure 3:**
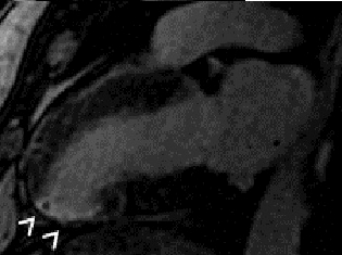
**Long-axis image on contrast-enhanced inversion-recovery magnetic resonance performed one month after the acute episode**. Wall thinning and transmural enhancement in the infero-apicolateral wall (arrowheads).

A submaximal exercise test on the sixth day was negative. An electrocardiogram on the seventh day (Figure 1B) showed a Q wave in inferior leads with apicolateral subepicardial ischemia. We did not detect any recurrence of the myocardial ischemia. A cardiac MRI performed one month after the acute episode identified a zone of myocardial necrosis and wall thinning, confirming the initial diagnosis (Figure 3).

## Discussion

Afibrinogenemia and hypofibrinogenemia (in its isolated type; not associated with dysfibrinogenemia) are congenital quantitative abnormalities of fibrinogen. They expose patients to a bleeding risk of variable severity depending on their phenotype. Thrombotic complications, however, have been reported in the literature in isolated cases. These cases were venous [1] or arterial thromboses and most occurred after administration of cryoprecipitates or fibrinogen. Nevertheless, some cases of spontaneous arterial thrombotic events have been reported (Table 1). The majority of them concerned peripheral arteries in patients with afibrinogenemia (where fibrinogen levels are lower than those in patients with hypofibrinogenemia, leading to a more significant rise in the circulating thrombin, but it muct be noted that afibrinogenemia also has a greater prevalence). To the best of our knowledge, only a single case of myocardial infarction has previously been reported, in a 27-year-old male patient with afibrinogenemia [2]. In our patient, in addition to the typical clinical, ECG and biological features which were coherent with the definition of acute myocardial infarction, the diagnosis was further confirmed by MRI. Nevertheless, the coronary thrombosis was not viewed by a coronary computed tomography angiography (which was unavailable) or by invasive explorations, which were problematic in view of the hemorrhagic risk. For the patient described by Kumar *et al*. [2], who was admitted two hours after pain onset, as well as for our patient, who was admitted seven hours after pain onset, the risk of bleeding that accompanies coronary angioplasty (and its adjunctive anticoagulant therapy) seemed to outweigh the expected benefit and it was therefore not indicated in either case. Also, because of the precarious balance of the process of hemostasis, thrombolytics and anti-thrombotic treatments could not be used. The use of these treatments classically contraindicated in fibrinogen deficient patients has nevertheless been described in cases of thrombotic complications, often in parallel with fibrinogen infusion [3-5,10].

**Table 1:** Cases of arterial spontaneous thrombotic complications in patients with fibrinogen deficiency reported in the literature

Reference	Age (years), sex	Fibrinogen deficiency	Thrombotic event
[6]	33 M	Afibrinogenemia	Iliac artery thrombosis and toe necrosis
[7]	37 F	Afibrinogenemia	Ischemic necrosis of the first left toe
[8]	37 F	Afibrinogenemia	Bilateral distal ischemia of fingers and toes
[1]	14 F	Afibrinogenemia	Ischemic necrosis of the right foot due to popliteal artery thrombosis
[3]	30 M	Afibrinogenemia	Ischemic lesions of the feet secondary to an occlusive lesion of the hypogastric artery and highly stenotic lesions of the iliac arteries (two episodes at three-year interval). After surgical bypass, asymptomatic occlusion of the bypass occurred at Day three.
[4]	22 M	Afibrinogenemia	Ischemic stroke in a context of aortic marastic endocarditis
[9]	21 F	Afibrinogenemia	Ischemia of the fifth toe then of the fourth toe
[2]	27 M	Afibrinogenemia	Inferior acute myocardial infarction
[5]	35 F	Hypofibrinogenemia	Renal infarction
	60 F	Hypofibrinogenemia	Toe ischemia by bilateral thrombosis of the anterior tibial arteries

F: female; M: male.

The increased risk of thrombosis in cases of fibrinogen deficiency could be explained by a rise in the level of circulating thrombin which is no longer inactivated by fibrin (previously known as antithrombin I factor) [3,10]. The thrombin, playing the role of platelet activator, favors the excretion of Von Willebrand factor from platelet alpha-granules. This later enhances platelet aggregation by binding to glycoprotein IIb/IIIa, leading to the formation of loose large thrombi [3]. Concerning the pathophysiologic mechanism, antiaggregant treatments can be effective in preventing the genesis of thrombotic events. Our patient received only aspirin; the patient described by Kumar *et al*. [2] received dual antiplatelet therapy (aspirin and clopidogrel). Neither of these two patients had an ischemic recurrence or a hemorrhagic complication.

## Conclusion

Arterial thromboses are at present recognized as possible, even if rare, manifestations of fibrinogen deficiencies. In addition to the particularity of their pathology, these thromboses raise diagnostic and therapeutic problems due to the precarious stability of the hemostasis balance in these patients.

## Consent

Written informed consent was obtained from the patient for publication of this manuscript and any accompanying images. A copy of the written consent is available for review by the Editor-in-Chief of this journal.

## Competing interests

The authors declare that they have no competing interests.

## Authors' contributions

FMZ realized the echocardiographic examination and was a major contributor in writing the manuscript. SM and JM assumed the clinical care and clinical follow-up of the patient and contributed to the analysis of hematological data. RT, NBM and MSM were responsible for the patient's care in the intensive care unit and contributed to the writing of the manuscript. HM and EM realized and interpreted the magnetic resonance imaging explorations. RM, who is Chief of the Service of Cardiology, assumed the main therapeutic decisions and revised the manuscript. All authors read and approved the final manuscript.
